# Navigating perinatal mental health care: migrant women’s experiences of support pathways and the role of digital resources

**DOI:** 10.1186/s13690-026-01989-x

**Published:** 2026-06-11

**Authors:** Areni Altun, Andrea Reupert, Melissa Oxlad, Rochelle Hine, Levita D’Souza, Malavika Kadwadkar, Jacqueline A. Boyle

**Affiliations:** 1 Eastern Health Clinical School, Monash University, 5 Arnold Street, Box Hill, Melbourne, VIC 3128 Australia; 2 School of Educational Psychology & Counselling, Monash University, Melbourne, Australia; 3 School of Psychology, The University of Adelaide, Adelaide, Australia; 4 School of Rural Health, Monash University, Bendigo, Australia

**Keywords:** Perinatal health, Mental health, Migrant women, Digital equity, Health equity

## Abstract

**Background:**

Migrant women experience disproportionately high rates of perinatal mental health difficulties, shaped by intersecting cultural, social, and systemic barriers. Despite the increasing role of digital technologies in healthcare delivery, their potential to support equitable perinatal mental health care remains underexplored and often fails to reflect the complex realities of migrant women’s lives. This study examined how women from migrant backgrounds in Australia navigate perinatal mental health care across clinical, community, and digital settings, with a particular focus on how digital tools interact with cultural, social, and structural factors to influence help-seeking and engagement.

**Methods:**

Qualitative data were collected through online focus groups and individual interviews with women from Chinese-, Arabic-, and Indian-language speaking backgrounds who had given birth in Australia. A semi-structured, culturally adapted interview guide explored women’s experiences of mental health support, barriers and facilitators to access, cultural beliefs, and the role of digital tools in finding/interpreting information. Interviews were conducted with interpreter support and analysed using reflexive thematic analysis to identify patterns across women’s narratives.

**Results:**

Three overarching themes, each with related subthemes, were developed: (1) *Accessing Perinatal Care* captured fragmented systems and difficulties navigating services that felt culturally unresponsive; (2) *Cultural and Socio-Emotional Context of Mental Health* reflected how stigma, traditional beliefs, and disrupted family networks shaped women’s emotional wellbeing, help-seeking, and comfort disclosing distress; and (3) *Support Seeking illustrated how women turned to digital platforms for* connection, and guidance but were constrained by language barriers, and varying levels of digital literacy and access.

**Conclusions:**

Migrant women navigate fragmented perinatal mental health pathways shaped by cultural, social, and structural barriers. Digital tools can support help-seeking, but only when designed to address cultural relevance, language needs, and digital inequities. Equity-focused, co-designed approaches are essential to ensure digital solutions genuinely enhance perinatal mental health care for migrant women.

**Supplementary Information:**

The online version contains supplementary material available at 10.1186/s13690-026-01989-x.

**Table:** 

Text Box 1. Contributions to the literature
• Provides novel population-level insight into how migrant women in Australia experience and navigate perinatal mental health across healthcare, family, community and digital settings.
• Demonstrates how fragmented services, limited cultural responsiveness, and stigma interact to delay help-seeking.
• Highlights the central role of social support, including family networks, peer communities, and digital platforms, in supporting perinatal mental wellbeing among migrant women.
• Extends evidence by showing how migration-related disruptions shape mental health needs during pregnancy and postpartum.
• Informs policy and service design by highlighting the importance of perinatal mental health support that brings together clinical care, social and emotional wellbeing, and digital resources.

## Background

Over the past five decades, global migration has increased steadily, with international migrants now comprising over 3.6% of the world’s population [1]. Today, more women live outside their country of birth and navigate maternity and health systems that are often distant from their cultural contexts and usual support networks. Migrant women often face unique challenges in the perinatal period (pregnancy, birth, and the first year postpartum), including systemic barriers, cultural dislocation, and unfamiliar care structures that are difficult to navigate [2, 3]. These, along with other socioeconomic factors, contribute to persistent disparities in perinatal mental health outcomes for women from migrant communities [4–6]. This study explores how migrant women navigate the transition to parenthood during the perinatal period with particular attention to the role of digital health in shaping their pathways to support. By examining how women engage with, interpret, and experience digital tools within broader social and structural contexts, the study aims to understand both the opportunities and limitations of digital health as a means of reaching more equitable perinatal mental health care.

Globally, perinatal mental health conditions affect up to 20% of women [7, 8]. However, women from refugee and migrant backgrounds face a high risk of perinatal mental health conditions, up to 40%, due to stressors experienced before, during, and after migration [7, 9]. These stressors include the challenges of resettlement, adapting to new health systems, building social networks, and balancing competing settlement priorities, which often take precedence over women’s health [10, 11]. Migrant women also experience barriers in navigating perinatal care, including language and health literacy challenges, difficulties navigating complex and unclear health system pathways, financial constraints, cultural beliefs that may conflict with mainstream practices, limited access to culturally responsive care, and increasing challenges associated with digital literacy and navigating online health platforms [12–15].

Perinatal care for women from migrant backgrounds involves a complex interplay between formal healthcare services (e.g., General Practitioners (GPs), hospitals, child and family health services) and informal or community-based support systems [16]. Increasingly, women also engage with digital health, including telehealth, web-based platforms, and mobile applications [17]. Digital technologies, such as telehealth, health information websites, and social media, have become increasingly important, offering information, connection, and access to services outside current settings [18]. However, health and digital literacy, access to devices and internet connectivity, language accessibility, trust in services, and cultural relevance influence the uptake of digital technologies [19].

Digital technologies have become integral to modern healthcare, providing accessible solutions for the prevention, diagnosis, treatment, and ongoing self-management of health conditions [3, 19, 20]. These tools potentially empower individuals to actively engage in their healthcare, offering convenient access to medical consultations, personalised health information, and self-monitoring resources that can enhance health outcomes and improve overall health experiences [21]. Digital resources can also help to establish a comprehensive network of community connections, particularly for individuals living outside urban centres and/or facing systematic marginalisation [22, 23].

The global burden of mental health conditions, including perinatal depression, highlights the urgent need for accessible and scalable management and support options [24]. While both psychotherapy and pharmacotherapy have been shown to be effective interventions for depression [25], many people face significant barriers to accessing in-person clinical services [26]. These barriers include a shortage of trained mental health professionals, lengthy waitlists, the stigma associated with help seeking and the high cost of care [26, 27]. While digital technologies offer an increasingly practical solution for migrant mothers during the perinatal period, significant barriers can limit their use and sustained engagement [20, 23]. Past negative experiences with healthcare systems may further discourage help-seeking, alongside concerns around privacy, security, and the reliability of unregulated online resources [14, 15]. Financial constraints, such as a lack of access to personal devices, unstable internet connectivity, or a lack of private spaces to engage with digital technologies, also remain key challenges [14, 15]. While these barriers limit the effectiveness of digital resources, addressing issues such as accessibility, cultural relevance, and trust could enable these tools to become a valuable resource for mothers during the perinatal period, a time when support and care are especially critical [28], and may have lifelong implications for infants and families.

Recent Australian studies have underscored the complex interplay between culture, service design, and social support in shaping migrant women’s perinatal experiences [3]. Evidence highlights the benefits of culturally responsive models of care, yet systemic barriers, such as language and cost, limited eligibility, and lack of culturally appropriate resources, continue to constrain equitable access and engagement [3, 29]. These studies also reveal that social isolation and disrupted support networks further amplify migrant women’s vulnerability, with many turning to digital and virtual forms of connection to bridge these gaps [29–31]. Consequently, there is an ongoing need for models of care that extend beyond traditional health settings and respond to the dynamic ways migrant women seek and experience support. However, while existing research has illuminated the importance of service accessibility and social support, less is known about how migrant women actively navigate, interpret, and engage with perinatal mental health care across clinical, community, and digital settings, and how digital resources intersect with these broader care journeys. The intersection of digital health, cultural identity, and structural inequities remains underexplored, particularly in understanding how women make sense of and utilise digital tools to support their perinatal wellbeing within the broader healthcare landscape.

In Australia, migrant women’s pathways to perinatal care are shaped not only by the structure of the healthcare system but also by global geopolitical factors, broad settlement experiences and community networks. This study extends previous research exploring five widely used, culturally inclusive online perinatal mental health resources [32] to explore how women from migrant backgrounds who have given birth in Australia navigate perinatal mental health care across clinical, community, as well as digital settings. Focusing in particular on digital resources allows us to examine their role, perceived utility, and integration into wider support pathways, while also exploring the strategies women employ and the challenges they face in accessing perinatal mental health care more generally. Understanding these pathways is essential for co-designing culturally responsive, accessible, and integrated models of perinatal care that address the unique needs of women from migrant backgrounds in Australia.

## Method

### Study design

This study, reported in accordance with the Consolidated Criteria for Reporting Qualitative Research (COREQ) guidelines [33], employed a qualitative research design using semi-structured interviews and reflexive thematic analysis to explore migrant women’s experiences with digital resources for perinatal mental health support [34]. This work extends previous research examining five widely used online perinatal mental health resources for women from migrant backgrounds [32]. While the prior study focused on accessibility and usability of these resources, the current study investigates how these digital resources are experienced and integrated within broader perinatal mental health support pathways. By focusing on these resources, we aim to understand their role, perceived utility, integration with other support services, and the strategies women use and barriers they encounter when seeking perinatal mental health care.

The study methodology was co-designed in collaboration with a Community and Consumer Involvement (CCI) Advisory Group to ensure cultural and linguistic appropriateness. The CCI Advisory Group included one woman from each of the three cultural groups in focus—Chinese-, Arabic-, and Indian-language speaking backgrounds. We use the terms ‘Chinese-‘, ‘Arabic-’, and ‘Indian-language speaking backgrounds’ to broadly refer to the range of languages commonly spoken in China, Arabic-speaking countries, and India, while acknowledging the significant linguistic diversity that exists within each of these groups. We selected these groups due to their demographic significance and documented health inequities. India is currently the leading country of birth for migrants to Australia, while Mandarin and Arabic are the most commonly spoken languages at home after English [35]. Women from these communities have also been shown to experience notable disparities in perinatal mental health outcomes and service access [36–40]. We recruited Advisory Group members through partnerships with multicultural health organisations, community networks, and maternal health services to ensure diverse representation across migration pathways (e.g., recent migrants, refugees), parity, English proficiency, and digital literacy levels.

The Advisory Group were central in shaping the study design, refining culturally sensitive interview guides, and advising on inclusive recruitment strategies. Advisory Group members attended research meetings and were reimbursed for their time and expertise in line with national consumer participation guidelines [41]. The Monash University Human Research Ethics Committee provided ethical approval in June 2024 (Project ID 43519).

### Participants and recruitment

Through a process of discussion and voting and in line with the most commonly spoken language, the research team and the Advisory Group identified three priority populations: women of Chinese, Arabic, and Indian-language speaking backgrounds. Participants were grouped by primary language (Arabic-, Mandarin-, and Indian language-speaking) to reflect the focus of the study. However, we acknowledge that these categories encompass substantial heterogeneity in cultural, regional, and linguistic backgrounds (e.g., differences across countries, dialects, and migration histories). Detailed information on participants’ specific regions or countries of origin was not collected, and as such, these groupings should not be interpreted as culturally homogeneous.

Eligibility criteria included being a woman aged 18 years or older, with a preference for residence in Victoria, Australia, who had migrated to Australia via skilled, family, or humanitarian visa pathways, who had given birth in the past five years and self-reported a history of perinatal mental health challenges. Participants were required to be conversant in Arabic, Mandarin, Hindi (or another Indian language), and/or English, and willing to engage with a professional interpreter if needed. While we aimed to recruit participants residing in Victoria, one participant was from South Australia and one participant from an Indian-language speaking background was born in Bahrain.

Women who required language support could choose to participate in individual interviews or language-specific focus groups, conducted in Arabic, Mandarin, or an Indian language such as Hindi. Professional interpreters were arranged through a certified health translation service accredited by the National Accreditation Authority for Translators and Interpreters (NAATI), and joined via secure video conferencing. In each case, only one interpreter per group or interview was used to maintain consistency and confidentiality.

The Advisory Group supported recruitment by reviewing and advising on the cultural appropriateness and clarity of recruitment materials (e.g., flyers, emails, social media posts), recommending trusted community organisations and platforms for dissemination, and advertising within their own networks. Several members facilitated introductions to community leaders and helped organise language-specific focus groups to ensure safe and culturally responsive environments. We employed a purposive sampling strategy to recruit 10 women from each cultural group, aiming to achieve diversity in age, length of time in Australia, visa pathways, English proficiency, and recency of childbirth. During recruitment, participants were asked whether they had ever experienced emotional or mental health challenges during pregnancy or in the year following childbirth. To ensure that participation did not place additional burden on women currently experiencing distress, potential participants were also asked whether they were presently receiving support or felt comfortable discussing their experiences. Women who disclosed current high levels of distress were provided with information on relevant support services and were not invited to take part until they felt ready to do so. We also used passive snowball sampling to reach women within each community, including those less familiar with health or digital services.

Interested women could contact the designated researcher (AA) directly or give permission to Advisory Group members to be contacted by AA via telephone or email. All participants received a plain language statement and provided written informed consent prior to participation. Women were informed that participation was voluntary and they could choose not to answer any question or stop the interview at any time. Each participant received a $30 AUD grocery voucher for their time.

During interviews and focus groups, participants were invited to reflect on their awareness of, and engagement with, perinatal mental health care in Australia, including whether, how, and where they sought information or support online, if at all. Discussions explored women’s experiences navigating care across different settings, including but not limited to digital platforms (e.g., online searches, apps, social media) and community-based sources of information.

### Data collection

We use the term “women from migrant backgrounds” to refer to women who were born overseas or who speak a language other than English at home. Informed by our consumer involvement approach, we did not collect refugee status, as this information can be sensitive, potentially retraumatising, and may have introduced legal or ethical complications beyond the scope of this study.

We used a combination of semi-structured individual interviews and focus groups. To ensure inclusive engagement, participants could choose their preferred format based on comfort, availability, and communication preferences. Using both formats enabled us to respect participants’ choice while also enriching the data by capturing both individual and group perspectives.

Prior to interviews and focus groups, participants were shown five pre-selected online perinatal mental health resources identified in our previous work [32]. They were then asked detailed questions about these resources, including how they located, evaluated, and used them (see Interview Guide in Supplementary Material). We acknowledge that this approach likely foregrounded discussion of digital resources, potentially shaping participants’ responses regarding trust, navigation, and perceived usefulness. At the same time, it allowed a focused exploration of digital supports while situating them within participants’ broader experiences of perinatal mental health pathways.

Examples of interview questions included: “What do you think can best help migrant women with their mental wellbeing before, during and after pregnancy?” and “Do you think there are any cultural beliefs that influence how women access online information or services?” Women were encouraged to share examples from their own or peers’ experiences, reflecting on both positive and challenging encounters to capture diverse perspectives across communities.

Data collection occurred between June and October 2024. Thirty women participated, across eight focus groups and three individual interviews. Focus group sizes ranged from two to four participants. The mean duration was 70 min for focus groups (range: 60–90 min) and 55 min for individual interviews (range: 45–65 min).

All sessions were conducted online via teleconference (Zoom) and facilitated by a trained qualitative researcher (AA). Where required, NAATI-accredited interpreters fluent in Arabic and Mandarin supported communication. Interpreters were briefed prior to each session and worked alongside the interviewer to ensure linguistic accuracy and cultural appropriateness (see Table 1). Translation was conducted in real time, with both the interviewer and interpreter actively engaged throughout the discussion.

All interviews and focus groups were audio-recorded and transcribed verbatim into English. For non-English sessions, transcripts were produced from the interpreter’s real-time English translation, which was recorded and then transcribed. Literal translation was prioritised where possible, and culturally significant terms were reviewed with the Advisory Group to ensure accurate interpretation. As transcripts were derived from interpreted English rather than original-language recordings, some nuance may have been lost. However, the credibility of the translation process was supported by researcher debriefing, detailed field notes, and peer review within the multilingual research team.

**Table 1:** Summary of participant engagement by language group and interview format

Cultural-Linguistic Group	Interview Type	Interpreter Used
Arabic-speaking	Focus Groups A (*n* = 2)	*\checkmark*
	Focus Groups B (*n* = 4)	X
	Focus Groups C (*n* = 4)	*\checkmark*
Chinese-speaking	Individual Interview	X
	Individual Interview	X
	Focus Groups A (*n* = 4)	X
	Focus Groups B (*n* = 4)	*\checkmark*
Hindi/Indian-speaking	Individual Interview	X
	Focus Groups A (*n* = 3)	X
	Focus Groups B (*n* = 3)	X
	Focus Groups C (*n* = 3)	X

### Data analysis

All transcripts were imported into NVivo version 14 to assist data management and facilitate the coding process. Data were analysed using reflexive thematic analysis [42], following an inductive and iterative approach to ensure interpretations remained grounded in participants’ accounts. AA independently completed initial reading, data familiarisation and open coding of the transcripts. Codes were subsequently organised into themes, each representing a coherent and recurring pattern of meaning. To support thematic development and contextualise findings, field notes and memos were also closely read.

While attention was given to potential variations across language groups and participant characteristics (e.g., English proficiency, time in Australia), the analysis was not designed to conduct formal comparisons between groups. Instead, the focus was on identifying patterns of meaning across the dataset, while noting where areas of convergence and divergence emerged across participants’ accounts.

Themes were iteratively revised in consultation with the wider research team and Advisory Group members, ensuring that the final interpretations accurately reflected participants’ experiences and culturally specific perspectives. To enrich analytic depth and support reflexive engagement with the data, a subset of transcripts was independently coded by a second researcher. This was not undertaken to achieve intercoder agreement or reliability, but rather to support reflexive dialogue, prompt critical questioning of emerging interpretations, and explore the range of meanings present in the data. Differences in interpretation were discussed collaboratively as a generative part of the analytic process, and insights were reviewed by our CCI advisory group to deepen the cultural grounding of our interpretations.

### Reflexivity

The intersecting identities and relationships between participants and researchers played a meaningful role in shaping this project [28]. Members of the research team brought relevant clinical, academic, and lived experience expertise in migrant health, perinatal mental health, and qualitative research. AA, a research fellow and clinical osteopath; JB, an academic gynaecologist and obstetrician; and RH, a social worker with extensive experience in women’s health and rural and mental health care, contributed diverse clinical and academic perspectives.

The broader research team included researchers from disciplines such as public health, psychology, digital health, and implementation science, who contributed to study conceptualisation, design, and interpretation. These included AR, LD, MO, and MK. While not directly involved in data collection, these team members played critical roles in shaping the study’s aims, providing analytical feedback, and ensuring alignment with health equity and culturally inclusive research principles. Importantly, the Advisory Group (MK, SS and LM) comprised women with lived experience of perinatal mental health challenges and migration. Regular reflexive team meetings were held to surface and discuss positionalities, potential biases, and interpretive influences. These discussions informed analytic decisions and ensured that the analysis remained grounded in participants’ experiences rather than researchers’ expectations.

AA, who led both data collection and analysis, brought important contextual insight to the study. As a woman born to migrant parents, AA shared cultural touchpoints with many participants. This shared background facilitated rapport-building and created an environment that was conducive to culturally safe engagement, enabling participants to comfortably discuss sensitive topics including trauma, mental health challenges and their experiences with digital resources during the perinatal period. While this closeness enhanced depth and richness in the data, it also required ongoing critical reflection to balance empathy and analytic distance. These relational dynamics contributed to an understanding of participants’ lived experiences while reinforcing the importance of reflexivity and methodological rigour throughout the study.

We acknowledge that no migration studies specialist was part of the team, which may have foregrounded a culturalist lens; this was mitigated through engagement with relevant migration studies literature [2] and iterative reflexive discussions to situate participants’ experiences within broader social and structural contexts.

### Findings

#### Participant characteristics

A total of 30 women participated in the study, with equal representation across the three language groups (10 participants per group). Most participants lived in metropolitan areas within Victoria, with a smaller subset residing in rural regions (*n* = 7). Ages ranged from 25 to 55 years, with the largest group falling within the 25–34 (*n* = 14) and 35–44 (*n* = 13) age brackets. Participants had lived in Australia for varying durations, from as short as six months to over 29 years. Educational backgrounds ranged from early high school qualifications to university degrees, with many having tertiary-level qualifications (*n* = 26). Most participants were married or in a relationship (*n* = 27), with family sizes ranging from one to four or more children.

While participants were grouped by language for analytic purposes, these categories do not reflect cultural or national homogeneity. All participants in the Chinese-language group were born in China. The Indian-language group was also predominantly India-born, with one participant born in Bahrain. The Arabic-language group was the most diverse in terms of country of birth, including participants born in Palestine, Iraq, and the Netherlands. This within-group diversity is reflected in Table 2 and underscores that language-based groupings should not be interpreted as culturally uniform populations. Additional demographic characteristics of the study participants are outlined in Table 2 (attached in a separate document).

**Table 2:** Demographic characteristics

Chinese-language speaking									
Participant	Age	Country of birth	Speaks English	Years lived in Australia	State	Metropolitan city or Rural regions	Education	Relationship status	Number of children
P1	35–44	China	Yes	8	VIC	Metropolitan city	Postgraduate degree	Married or in a relationship	2–3
P2	25–34	China	Yes	6	VIC	Metropolitan city	Postgraduate degree	Married or in a relationship	2–3
P3	35–44	China	Yes	16	VIC	Metropolitan city	Bachelor’s degree	Married or in a relationship	1–2
P4	25–34	China	Yes	10	VIC	Metropolitan city	Bachelor’s degree	Married or in a relationship	1
P5	35–44	China	Yes	22	VIC	Metropolitan city	Postgraduate degree	Married or in a relationship	2–3
P6	25–34	China	Yes	11	VIC	Metropolitan city	Postgraduate degree	Married or in a relationship	2–3
P7	25–34	China	No	10	VIC	Metropolitan city	Postgraduate degree	Married or in a relationship	1
P8	25–34	China	No	15	VIC	Metropolitan city	TAFE/vocational degree	Separated or divorced	2–3
P9	35–44	China	No	16	VIC	Metropolitan city	Bachelor’s degree	Married or in a relationship	2–3
P10	35–44	China	No	19	VIC	Rural region	Postgraduate degree	Separated or divorced	1

Indian-language speaking									
Participant	Age	Country of birth	Speaks English	Years lived in Australia	State	Metropolitan city or Rural regions	Education	Relationship status	Number of children
P1	25–34	India	Yes	10	VIC	Metropolitan city	Postgraduate degree	Married or in a relationship	1
P2	35–44	India	Yes	10	VIC	Rural region	Postgraduate degree	Married or in a relationship	1
P3	35–44	India	Yes	7	VIC	Metropolitan city	Bachelor’s degree	Married or in a relationship	2–3
P4	45–55	India	Yes	7	SA	Metropolitan city	Postgraduate degree	Separated or divorced	1
P5	25–34	India	Yes	8	VIC	Rural region	Postgraduate degree	Married or in a relationship	1
P6	25–34	India	Yes	5	VIC	Metropolitan city	Postgraduate degree	Married or in a relationship	1
P7	25–34	India	Yes	5	VIC	Metropolitan city	Bachelor’s degree	Married or in a relationship	1
P8	25–34	India	Yes	7	VIC	Metropolitan city	TAFE/vocational degree	Married or in a relationship	2–3
P9	45–55	India	Yes	15	VIC	Metropolitan city	Postgraduate degree	Married or in a relationship	2–3
P10	35–44	Bahrain	Yes	11	VIC	Metropolitan city	Bachelor’s degree	Married or in a relationship	1

Arabic-language speaking									
Participant	Age	Country of birth	Speaks English	Years lived in Australia	State	Metropolitan city or Rural regions	Education	Relationship status	Number of children
P1	25–34	Palestine	No	7.5 months	VIC	Rural regions	Bachelor’s degree	Married or in a relationship	2–3
P2	25–34	Palestine	No	6 months	VIC	Rural regions	Bachelor’s degree	Married or in a relationship	1
P3	35–44	Palestine	No	9 months	VIC	Rural regions	Bachelor’s degree	Married or in a relationship	2–3
P4	25–34	Palestine	No	8 months	VIC	Rural regions	Bachelor’s degree	Married or in a relationship	1
P5	25–34	Palestine	No	4 months	VIC	Metropolitan city	Bachelor’s degree	Married or in a relationship	2–3
P6	35–44	Netherlands	Yes	17 years	VIC	Metropolitan city	Postgraduate degree	Married or in a relationship	1
P7	35–44	Iraq	No	9 years	VIC	Metropolitan city	Year 12	Married or in a relationship	1
P8	35–44	Iraq	No	29 years	VIC	Metropolitan city	Year 12	Married or in a relationship	4 or more
P9	35–44	Iraq	No	13 years	VIC	Metropolitan city	Grade 11 or less	Married or in a relationship	2–3
P10	45–55	Iraq	No	29 years	VIC	Metropolitan city	Grade 11 or less	Married or in a relationship	4 or more

### Themes

Three overarching themes, and related subthemes captured migrant women’s experiences of perinatal mental health care in Australia (Table 3). Theme 1 *Accessing Perinatal Care* described women’s experiences with fragmented services, limited attention to maternal mental health within routine care, and their preferred ways of engaging with services and support networks. Theme 2 *Cultural and Socio-Emotional Context of Mental Health* reflected how stigma, cultural expectations, and disrupted support networks shaped wellbeing and help-seeking. Theme 3 *Support Seeking* illustrated how women turned to digital platforms to navigate information, connect with peers, and rebuild community in the absence of familial supports. Overall, themes were broadly shared across participants, although some variations were observed in how experiences were described, particularly in relation to English proficiency, familiarity with services, and access to digital resources.

**Table 3:** Summary of themes and sub-themes

Theme	Sub-theme
1. Accessing perinatal care	System gaps and fragmented care
	Lack of prioritisation for maternal mental health
	Preferred methods of connection
2. Cultural and Socio-Emotional Context of Mental Health	Stigma and mental health disclosure
	Negotiating different cultural practices
	Cultural Inclusivity in healthcare
	Motherhood without a village
3. Support Seeking	Navigating digital health information
	Digital networks as spaces of care and connection

#### Theme 1: accessing perinatal care

This theme captures how women encountered a fragmented and often confusing perinatal care system, highlighting three interconnected subthemes. First, *system gaps and fragmented care* left women navigating inconsistent information, unclear referral pathways, and limited communication between providers. Second, the *lack of prioritisation for maternal mental health* meant that women’s emotional needs were often overlooked in favour of infant-focused care, leaving many unaware of symptoms or support options. Third, women described their preferred methods of connection, emphasising the importance of trusted interpersonal contact – whether through family, peers, or phone-based support.

##### Sub-theme: system gaps and fragmented care

Many women described navigating perinatal care as overwhelming, citing fragmented services, conflicting information, and unclear referral pathways. These systemic challenges often left participants feeling unsupported, particularly when in-person care was limited to referrals rather than guided assistance. As one participant explained: “Sometimes the staff… they won’t give too much emotional support. They just say, ‘Oh, you need to find a psychiatrist or other professional.’ But if I could find them, why would I call you?” (Chinese-speaking 1:1 interview).

While these challenges were described across all groups, their severity and nature varied, particularly in relation to English proficiency and familiarity with the Australian health system. This was illustrated by accounts from some Arabic-speaking participants, some of whom described uncertainty about where to go for help and a tendency to rely on community contacts or Arabic-language websites. For some, even accessing online information was difficult due to limited literacy, as one participant stated: *“Some of them… don’t know how to go on internet… do search and find what they’re looking for because they don’t even read or write in Arabic”* (Arabic-speaking FGC). In contrast, many participants from Chinese and Indian/Hindi-speaking backgrounds – most of whom were tertiary educated and fluent in English – were able to search independently but still struggled with inconsistent guidance, unfamiliar systems, and unclear referral pathways.

Participants also highlighted gaps in communication across care providers. Despite frequent interactions with obstetricians, GPs, and midwives, women were often unaware of available supports, particularly online resources. As one participant from a Chinese-speaking background (FGB) explained: “I’ve visited my obstetrician, my GP, my midwife… not a single one of them actually mentioned [perinatal] websites. Is it possible for the government to communicate with medical practitioners to actively advocate for these resources?”.

One participant reflected on her second pregnancy, noting that support for mothers beyond the first pregnancy was often overlooked. She described receiving “hardly any resources” because “you’re a second-time mum, you know everything,” emphasising that “each delivery is unique… information should be given whether first-, second-, or third-time mum.” (Indian-speaking participant FGC).

For many women, recognition of their need for support occurred only by chance, as systematic screening for perinatal mental health concerns was limited. One participant from a Chinese-speaking background (FGA) reflected that it was only during a six-week appointment that she realised she was struggling, noting: “Most people wouldn’t know [they need help] until someone points it out.” Language barriers and the lack of translated materials compounded these challenges. Another participants remarking that having leaflets available in Arabic at GP clinics “would have definitely attracted my attention.” (Arabic-speaking participant FGB).

Financial constraints and long wait times further fragmented access to care. As one participant noted: “Financial condition also would be very important… if the service can be bulk billed, it will… reduce the barrier for the migrant mum to access those resources” (Chinese-speaking 1:1 interview).

##### Sub-theme: lack of prioritisation for maternal mental health

Participants consistently reported that maternal mental health was often deprioritised in perinatal care, with clinical interactions focused predominately on the baby rather than the mother’s wellbeing. Many described feeling overlooked during routine appointments, where their emotional or mental health needs were rarely discussed. One participant from an Indian speaking background (FGC) reflected: “[when] a pregnant lady goes to the check-up, the nurses always talk about the baby… but it would be really great if they asked about us too”. Similarly, another participant noted that follow-up care focused solely on the infant “After the first two weeks, all other MCH [maternal child health] appointments were for the kid. There wasn’t any follow-up for the mother (Indian-speaking FGC).

Women often only recognised their own struggles in hindsight. One participant shared: “When I had my first born… we were both exhausted… we didn’t know we need help” (Chinese-speaking FGA). Others were unaware of their own symptoms stating: “So many times the mums don’t even know that they’re going through postnatal depression or anything. They may not be aware,” (Indian-speaking FGB).

##### Sub-theme: preferred methods of connection

Women described diverse preferences for accessing perinatal mental health support, balancing the convenience of online information with a desire for personal, trusted connections. Across focus groups, participants described turning first to family for reassurance and guidance for both emotional wellbeing and maternal health decisions. A participant from an Arabic-speaking background (FGB) shared: “If I want advice I go to my mum… even when I’m in Australia I still call them and ask them many advice”. Likewise, health decisions were often guided by those seen as “senior” family members, and whose lived experience and authority carried weight in shaping perinatal and parenting choices: “In our community we all follow our family members or whatever experiences, our senior sisters or brother, like sister-in-law, we talk with them… they have the experience, so we value their guidance for our health decisions” (Indian-speaking FGA).

While family networks remained central, women also used digital resources for quick guidance or reassurance when professional help was not immediately available. One participant noted: “The first thing I go [to is] the internet, it’s easier, faster…[it] guides me until I’ll get medical help” (Arabic-speaking FGA). However, online resources were rarely viewed as sufficient on their own as one participant shared: “I would rather call somebody if I need help rather than searching the website…” (Chinese-speaking 1:1 interview).

Some described positive experiences with telephone counselling, which they regarded as a bridge between impersonal online information and face-to-face interaction: “I actually spoke with one of the counsellors… we had a couple of sessions over the phone, which was great” (Indian-speaking 1:1 interview). Others preferred interactive peer workshops that allowed collective learning and sharing: “If they make [a] workshop with migrant [women], pregnant or having had a baby… this give us idea more than the website… this is the best for us” (Arabic-speaking FGA).

For women experiencing heightened stress, particularly in the postpartum period, immediacy and locality were critical. One participant described how a friend “didn’t have the time and energy to go on a website and ask for help… if she had access to a contact person she could reach out to… every mum needs a very immediate local help close to her, especially in emergency time” (Indian-speaking FGA). Participants suggested practical ways to improve access, including translated pamphlets and posters with QR codes in hospitals, so information “goes directly to the website” (Indian-speaking FGB).

#### Theme 2: cultural and socio-emotional context of mental health

This theme reflects how cultural norms, social expectations, and disrupted support networks shaped women’s emotional wellbeing and decisions about seeking help. The subtheme of s*tigma and mental health disclosure* revealed how shame, fear of judgement, and ideals of maternal strength constrained women’s ability to acknowledge distress. *Negotiating different cultural practices* captured the tensions women navigated between traditional beliefs and Western healthcare advice, particularly around confinement, breastfeeding, diet, and infant care. The subtheme of *cultural inclusivity in healthcare* illustrated gaps in cultural understanding among providers, leaving women feeling unseen or misunderstood when their practices were dismissed or not accommodated. Finally, *motherhood without a village* highlighted the emotional and practical consequences of migration, including isolation, loss of intergenerational care, and the compounding impacts of transnational grief.

##### Sub-theme: stigma and mental health disclosure

Across all groups, women described mental health as a sensitive and, at times, taboo topic, one that was often met with discomfort or silence, regardless of cultural background. While participants recognised that stigma around mental health exists more broadly in society, many felt that cultural expectations and community norms intensified these challenges after migration. One participant explained: “You don’t want people to think you are weak… so you just keep it to yourself” (Arabic-speaking (FGA).

Several participants reflected that stigma persisted even after migration, particularly within close-knit communities. As one participant observed: “Mental health still would be not an open topic in China, so around some Chinese population here, I think other friends I know still have some stigma to talk about it” (Chinese-speaking 1:1 interview).

Within some communities, motherhood was idealised as a period of fulfilment and resilience, leaving little room to express vulnerability. Mothers were expected to “just cope” without complaint, as one participant described: “In our culture … we have pressure on the mother not to [ask for help]… It’s normal to hear, ‘You are a mother, you have to just go on… we all have this journey.’ Sometimes you just want a little bit of support, but we don’t have this in our culture actually.” (Arabic-speaking FGB).

Likewise, expressions of sadness or anxiety were sometimes interpreted as a sign of spiritual deficiency, or ingratitude as one participant shared:

“Sometimes mistakenly, you know depression or anxiety are classified as a lack of gratefulness, gratefulness towards God… traditionally if you’re just saying, you know, ‘I’m feeling low’ or ‘I’m feeling depressed’, you are just told ‘Okay, just be grateful for everything you’ve got in life,’ and that’s not a very helpful attitude” (Arabic-speaking FGB).

Some participants described concealing distress due to cultural expectations to appear strong. One participant explained that she would “keep smiling so people don’t ask questions” while “appearing fine, even if you are feeling sad” (Chinese-speaking 1:1 interview).

The expectation that new mothers should feel only joy after childbirth compounded this silence, as acknowledging sadness or anxiety could be seen as incompatible with being a “good mother”. One participant shared: “If you’re feeling sad and anxious, there might be cultural reasons where you don’t feel it’s okay to feel sad and anxious, because you’ve just had a baby and that should be good news” (Arabic-speaking (FGB).

Underlying these fears was a deep anxiety about how disclosure might affect their role as caregivers. Though less commonly expressed, some women highlighted that admitting to mental health difficulties could invite judgement about their parenting capacity: “If you tell people, they might think you can’t look after your baby… and then what will happen?” (Arabic-speaking FGB).

##### Sub-theme: negotiating different cultural practices

Participants frequently described the perinatal period as a site of negotiation between their *own* culturally grounded practices and the expectations of the Australian healthcare system. Despite differing cultural practices across groups, many women shared a common experience of feeling torn between family guidance – shaped by traditions around postpartum recovery, diet, infant care, and broader cultural norms – and the often contrasting advice provided by healthcare professionals. These tensions were particularly evident in relation to postpartum care practices. One participant described: “The Chinese traditional postnatal, prenatal process is very different from the Western culture… The first challenge you actually face is what your Mum and Grandmum tell you – it’s very different from what nurses and doctors tell you” (Chinese-speaking FGA).

These competing and at times contradictory messages were particularly evident in relation to diet and postpartum recovery. Cultural beliefs, such as avoiding certain foods, were often absent from mainstream medical advice, leaving women unsure how to reconcile both perspectives. One participant reflected: “In Asian culture, my mum always used to tell me to avoid a lot of papaya because it generates heat… These kinds of beliefs are missing on professional websites. Unless there is a scientific reason, I wouldn’t immediately believe them, but at the same time I would listen to my Mum. Websites should acknowledge these practices while giving medical reasoning, so women can make their own informed choice” (Indian-speaking FGA).

Similar pressures were described in relation to breastfeeding. One participant explained: “The mums and the mother-in-law, all the time just to try many times [but] the babies refuse to take the [breast], but in our culture you want to breastfeed the baby… it makes a pressure” (Arabic-speaking FGB).

For some, these negotiations generated strain within families, where differing intergenerational expectations around newborn care often clashed:

“When my baby caught a cold, my mother-in-law talked about a lot of traditional Chinese beliefs. I said, ‘I’ve read all this information online from Royal Children’s Hospital,’ but she replied, ‘You can read those, but you still need to believe traditional Chinese ways from years of experience.’ Sometimes it’s really hard to look after the baby together, especially when we’re open to both Chinese and Western ways, but they’re not always open-minded” (Chinese-speaking FGA).

Such moments of disagreement added strain to an already emotional and demanding period as one participant noted: “They’re already emotional, looking after a newborn, and then they have to deal with a barrier created with their own Mum or parents. That’s hard.” (Chinese-speaking participant FGA).

Others noted that mainstream parenting advice often failed to reflect the realities of culturally diverse families. One participant from an Arabic-speaking (FGB) background explained: “it would be helpful to have resources that merge cultural and Western perspectives, with websites offering “different perspectives… that would be more relevant to a parent like me.” Accordingly, many participants expressed the need for culturally tailored and bilingual resources that could help them mediate these differences and avoid conflict with family members. A participant from a Chinese-speaking background (FGA) suggested:

“I feel like a certain section on the website or even just a small handout available in Chinese may be helpful to show the elders, say ‘Look, this is from a reputable website.’ … It’s about how we do confinement in a modern way. Not the traditional way, no shower, no drinking cold water for a whole month. That’s quite unrealistic”.

##### Sub-theme: cultural inclusivity in healthcare

For many recently arrived participants, parenting within a new cultural environment brought both emotional and practical challenges. Participants described the difficulty of reconciling their cultural values with those of Australian society, particularly when raising children in bicultural settings. A participant from an Arabic-speaking (FGB) background reflecting: “The most important thing for me… is how we can raise our children in this different culture. I don’t want them to feel different…so I want advice from people here, especially because I just came a few months ago”. These tensions were heightened by a lack of culturally informed guidance from healthcare providers. Standardised tools such as growth charts and developmental milestones were often perceived as not reflecting the diversity of different population groups. One participant described:

“Indian babies tend to be smaller compared to Aussie babies… For their genetics, the baby is of normal size, but since all babies are measured in one single frame, they tend to judge the baby as too small or too big, which adds a lot of stress” (Indian-speaking 1:1 interview).

Participants emphasised that cultural inclusivity in care extended beyond providing translated materials; it required healthcare professionals who could engage with and respect diverse cultural traditions. One participant from a Chinese-speaking background (FGB) explained: “It is better if the person you talk to understands our traditions… otherwise you have to explain so much”. When cultural understanding was absent, women felt unseen and fatigued by the constant need to justify their practices, reinforcing a sense of disconnection from the healthcare system.

##### Sub-theme: motherhood without a village

For many participants, migration profoundly reshaped the social supports they typically relied on during the perinatal period. The loss of these familiar networks of care intensified feelings of vulnerability and emotional strain. One participant highlighted:

“In our culture, at least the mother or mother-in-law can come and take care of the new mother and baby. But because we are migrants, most of the time it does not happen due to visa restrictions, or they cannot stay longer because they have to go back home” (Indian-speaking FGB).

Without familiar support, women described the transition to caring for a newborn as overwhelming and emotionally destabilising: “After that, you suddenly had a mother taking care of you for six months, and now suddenly you’re alone taking care of the baby. That can be extremely overwhelming because you’ve been so dependent on them… that’s when you would need help, [for] mental health especially” (Indian-speaking FGB). Global conflicts, humanitarian crises, and transnational grief reverberated deeply within diasporic communities, compounding emotional distress during an already vulnerable time. One participant from an Arabic-speaking background (FGB) shared:

“A lot of women are experiencing vicarious trauma within the diaspora, whether we’re from Palestine, Iraq, Iran… My friend, who’s pregnant with twins, has been inconsolable because of what’s happening in Lebanon. These feelings of intense sadness and despair can’t be healthy during pregnancy, but I couldn’t see any mention of this on maternal health websites”.

#### Theme 3: support seeking

This theme explores how women from migrant backgrounds navigated and constructed support systems without always having formal guidance. In the subtheme *navigating digital health information*, women described both the opportunities and overwhelm of online health content, emphasising the difficulty of identifying credible sources amid conflicting advice. In *digital networks as spaces of care and connection*, women highlighted the importance of culturally familiar online communities (particularly in their first language) as sources of reassurance, collective wisdom, and emotional support. These digital spaces sometimes functioned as informal ‘villages’ that helped fill gaps left by fragmented health systems and absent family networks.

##### Sub-theme: navigating digital health information

Participants described being overwhelmed by the abundance of online information, with “so much conflicting information” (Arabic-speaking FGB), creating confusion and uncertainty about what and who to trust. Likewise, the digital environment was simultaneously a space of opportunity and overload. As one participant reflected: “I tried to find a few things on Google and sometimes I find more than one opinion… sometimes these opinions are opposites to each other, and you are lost” (Arabic-speaking FGA).

While many participants actively searched for information online, few felt confident distinguishing reliable advice from opinion. Some described a gap between information access and meaningful understanding: “I do not know about these kind of websites where parents get help… people just say you’re going to get cranky after delivery, but they don’t say why, or how to cope with it when you are in that situation” (Indian-speaking FGC).

For others, professional endorsement was key to navigating this uncertainty as one participant described: “Sometimes too much information makes you puzzled… the correct information should come from the correct group of people. My reference was the GP or the community, instead of Google” (Indian-speaking FGA). Yet, identifying credible sources depended on understanding digital language, and even knowing the correct search terms: “If you just put the word ‘pregnancy,’ lots of information comes up. But correct information from the right people is what’s really helpful” (Indian-speaking FGA).

##### Sub-theme: digital networks as spaces of care and connection

Women across all groups turned to digital networks to supplement fragmented formal care, but the type of platforms they relied on differed markedly according to cultural and linguistic needs. These digital spaces offered immediacy, shared language, and a sense of belonging. As one participant from an Arabic-speaking background noted: “That is really nice to find a community online and to see that, okay, I’m not the only one who struggles with this” (Arabic-speaking FGB).

While digital engagement was common across groups, the platforms used and level of reliance varied depending on language preferences, trust, and familiarity with digital tools. For women with limited English, digital networks in their first language served as accessible entry points for both advice and solidarity as one participant described: “I just imagine for women with limited English… I would just ask a question in the WeChat group… the quickest, easiest way… there are hundreds of mums there” (Chinese-speaking FGA). Another participant from a Chinese -speaking background shared how these networks often became the primary source of prenatal support stating: “…all the supports that I had mostly come from the WeChat mums’ group” (Chinese-speaking FGA).

Culturally familiar perspectives shared by trusted professionals were especially valued as one participant shared: “I also follow some OBGYNs (Obstetricians and Gynaecologists)… because they speak in Arabic and then I can hear it from an Arabic or an Islamic perspective” (Arabic-speaking FGB). However, other participants from Indian-language speaking backgrounds described a preference for ‘word-of-mouth’ referral rather than digital platforms suggesting:

“I think [support] would mostly have to do with social circles, like a women’s group, mother’s group or like friends referring to friends. Because I think we’re more likely to, at least I am more likely to take information coming from a person that I trust or I know.” (Hindi-speaking Focus Group B).

However, these platforms also carried risks. The open nature of some social media spaces could amplify misinformation or tensions. One participant from a Chinese-speaking background explained: “Anyone can post, so you don’t know whether it’s true or not… my mother-in-law uses this a lot… ‘These people say this, so I’m right.’ It’s a little, you need to follow my suggestion… it’s tricky” (Chinese-speaking FGA).

Despite these limitations, most participants engaged with social media as a key touchpoint for information: “If it pops up on my Instagram or Facebook… if that information is relevant to me I would click on it” (Indian-speaking FGB). Culturally specific apps also supported community building and continuity of cultural practices. One participant from a Chinese-speaking background shared: “As a new immigrant from China, there is a popular app called Red… it has everything… sometimes people post mother groups on there. So I joined the mother group from this app” (Chinese-speaking 1:1 interview).

Over time, these digital communities often evolved into informal “villages” of care. One participant described how, in WeChat mothers’ groups, discussions about “feeling depressed… or even family violence” created a space for support (Chinese-speaking FGA).

## Discussion

This study provides insights into the experiences of women from migrant backgrounds navigating perinatal health care in Australia, including the role of digital health. Key areas included fragmented healthcare and support pathways; stigma surrounding mental health within communities, which affected disclosure and help-seeking; culturally discordant advice, which led to confusion; and digital and community supports as opportunities for obtaining information and partially substituting for absent family networks. Importantly, participants identified social support, whether through family, peer groups, or digital platforms, as central to their wellbeing, highlighting the need for integrated approaches that bridge clinical care with culturally relevant community and social supports. For some participants, digital resources were used not only as supplementary tools but as important and often first-line sources of information and connection, influencing how they recognised, interpreted, and responded to perinatal mental health needs.

Participants highlighted significant systemic gaps in perinatal care. Despite frequent contact with healthcare professionals including GPs, midwives and maternal child health nurses, mental health issues were often missed, minimised, or deprioritised. Many women experienced fragmented care, inconsistent advice, and unclear referral pathways, which left them feeling unsupported. These gaps were intensified by structural barriers including language challenges, a limited number of culturally responsive practitioners, and the scarcity of translated or culturally adapted resources, factors well documented in prior research as intensifying inequities in access and quality of care [43–45]. In response to fragmented services, women often turned to digital resources to navigate care pathways; however, inconsistent information, lack of professional endorsement, and varying digital literacy limited their effectiveness as standalone solutions [20, 46–48].

Fragmentation was also reflected in the way care was organised and delivered. Specifically, participants described perinatal services as routinely being overshadowed by an institutional focus on the infant. Postnatal checks were described as largely oriented toward the baby’s growth, feeding, and development, with minimal inquiry or attention to maternal emotional wellbeing. This structural prioritisation of infant health over maternal mental health has been observed in other high-income settings, where perinatal care models often insufficiently integrate maternal wellbeing into routine care [49–54]. The under-recognition of maternal distress represents not only a clinical gap, but a systemic disconnection between women’s needs and service delivery. For many participants, this absence of consistent, woman-centred mental health support contributed to their reliance on digital resources to seek information, validation, and direction, positioning digital platforms as informal extensions of care rather than integrated components of the health system.

Although stigma is not unique to women from migrant backgrounds [55], stigma emerged as a consistent barrier to care across cultural groups. Women described internalising expectations to appear strong and coping, even when struggling, and feared being judged as unfit, ungrateful, or inadequate mothers [56]. While participants did not explicitly describe fears of child removal, this apprehension about being seen as an unfit mother contributed to women’s hesitancy to seek help or disclose distress. These concerns were intensified by cultural taboos surrounding mental illness and broader societal ideals of motherhood [56, 57]. As a result, stigma was not only a barrier to help-seeking, but a mechanism shaping how and when distress was disclosed, with many women delaying or avoiding engagement with formal services. This pattern of concealment and delayed disclosure has been consistently reported in international literature. Ford et al. (2019) found that fear and stigma were the strongest barriers to disclosing perinatal mental health problems in primary care, outweighing practical challenges such as appointment access [58]. In the UK, up to 70% of women hide or minimise symptoms due to stigma and limited awareness, contributing to under-recognition and under-treatment of mental distress [59, 60].

Stigma, both cultural and societal, remains a central barrier to timely identification and support for perinatal mental health. For some women, digital platforms provided a private and less stigmatising avenue to explore mental health concerns, enabling anonymous information-seeking, peer connection, and recognition of distress. In this way, digital spaces may help to lower initial barriers to engagement by offering forms of support that feel safer and less exposing than formal services. However, these benefits were not universal. Consistent with broader literature, many digital resources were perceived as culturally misaligned and did not fully resonate with women’s lived and living experiences or cultural frameworks [14, 61, 62]. As a result, while digital tools could facilitate initial engagement, they did not consistently provide greater disclosure or sustained support. Furthermore, stigma and limited mental health literacy further complicates help-seeking, with women often struggling to recognise symptoms or discern the relevance of available supports [56, 57]. Taken together, these findings suggest that interventions to improve perinatal mental health outcomes for women from migrant backgrounds must move beyond accessibility alone to actively address stigma, promote culturally grounded mental health literacy, and create safe, trusted spaces – both digital and in-person – where women feel permitted to disclose distress without fear of judgement.

Women experienced tension when navigating traditional cultural practices and Western medical advice. Women reported receiving conflicting guidance from family elders and clinicians on postpartum care, nutrition, and infant feeding. While family advice carried emotional and cultural significance, clinical recommendations often differed, leaving women caught between competing expectations. This dissonance created stress and, at times, intergenerational conflict, particularly when women’s choices were perceived as deviating from cultural expectations. Similar tensions have been reported elsewhere, where culturally discordant advice can undermine trust in healthcare providers and discourage disclosure of distress [63, 64]. In this context, women were not simply choosing between two sources of advice, but actively negotiating care in ways that preserved cultural identity, family relationships, and perceived safety, often in the absence of culturally responsive guidance from health services. This process of negotiation has been observed in other migrant populations. For example, Zhi et al. (2024) found that Chinese migrant mothers in Australia sought to integrate both cultural and Western evidence-based practices, yet tensions commonly arose when their preferences conflicted with the views of mothers, mothers-in-law, or peers [65].

These tensions were not resolved within digital spaces. Rather than functioning as neutral sources of information, many digital resources reflected dominant Western biomedical perspectives, which did not align with women’s cultural views or practices. As a result, digital platforms became an additional site of negotiation, where women were required to reconcile competing forms of knowledge across family, clinical, and online domains. Participants suggested that culturally inclusive resources, those that acknowledge traditional practices while explaining biomedical rationales, may help to support more informed and culturally meaningful decision-making. This highlights the need for digital and clinical interventions that move beyond translation alone, toward the integration of culturally grounded knowledge systems within perinatal care. Perinatal screening tools, assessment frameworks, and referral pathways that fail to account for these differences may further emphasise barriers to early identification and support [56, 57].

Family networks particularly partners, mothers, and mothers-in-law were often the first and most trusted sources of support. These relationships provided emotional reassurance and practical guidance, helping women interpret their distress. Migration-related disruptions, such as visa restrictions preventing extended family visits, often left many women without their primary support networks during the postpartum period. Women described this abrupt withdrawal of familial support as both isolating and overwhelming. These findings align with a growing body of international evidence demonstrating that migration-related separation from family and familiar cultural networks contributes to social isolation and loneliness among migrant mothers [66–68]. For example, a Canadian study found that social isolation was closely tied to the loss of established family and cultural supports, while loneliness reflected a deeper sense of disconnection and lack of belonging in a new social environment [67]. Similar patterns have been reported in studies from the UK and Australia, where women from migrant backgrounds describe the absence of extended family as a critical gap in postpartum support, contributing to emotional distress and challenges in adapting to motherhood [69–71]. In this context, digital platforms functioned as surrogate support networks, enabling women to reconstruct forms of social connection and reassurance otherwise disrupted by migration. Digitally mediated resources have the potential to enhance community-based supports to strengthen the social scaffolding that underpins perinatal wellbeing.

Participants actively sought both digital and in-person connections to make sense of their experiences and find reassurance. Digital platforms, culturally specific apps, and online peer groups provided accessible spaces for sharing and support, often in women’s first languages. However, engagement with these resources was shaped by varying levels of digital literacy, confidence, and familiarity with online health information. In contrast, in-person support networks, such as community workshops or culturally tailored parenting groups, offered relational continuity and validation. These social spaces also facilitate knowledge exchange, helping women recognise perinatal mental health symptoms, normalise their experiences, and identify when professional care is needed [72]. However, while digital resources offered important avenues for connection both online and in-person, information was not equally accessible or beneficial for all women. Variations in health and digital literacy, language proficiency and trust shaped how women engaged with these platforms. For some, digital information – sometimes unverified and inconsistent – created confusion, requiring them to navigate competing and sometimes contradictory advice without clear markers of credibility or cultural relevance.

Our findings illustrate that migrant women’s perinatal experiences are deeply shaped by the interplay between culture, social structure, and migration histories. Participants described how the absence of culturally anchored ‘villages’ of care, the emotional burden of distant crises, and the need to recreate collective support through online communities shaped both their wellbeing and help-seeking. These accounts show that women’s needs cannot be understood solely at the individual level but reflect collective identities, transnational responsibilities, and culturally integrated expectations of care [64]. Within this context, cultural humility, which recognises difference, avoids assumptions, and invites partnership, becomes essential in supporting women from migrant backgrounds with both in-person and in digital spaces and resource. Attention to these structural factors may help address systemic inequities embedded within perinatal care systems and associated digital supports, particularly where services fail to adequately reflect the cultural and social realities of migrant women [73–75]. Together, these findings highlight that perinatal wellbeing is not only shaped by access to services, but by the ability to reconstruct meaningful, culturally grounded networks of care in the context of migration.

### Limitations

Several limitations should be noted. Although this study included participants from Chinese-, Arabic-, and Indian-speaking backgrounds, it does not capture the full diversity of women from migrant backgrounds experiences within Australia. While data on characteristics such as length of stay in Australia were collected, this study did not specifically examine within-group differences (e.g., visa status, duration of residence, acculturation, or socioeconomic position). These factors are likely to shape experiences of accessing perinatal mental health support and warrant more focused investigation in future research. Similarly, as we did not collect detailed data on participants’ specific countries or regions of origin, we were unable to explore within-group differences. We also note that terminology used to describe migrant populations can vary, and while we aimed for consistency, these terms encompass diverse experiences and identities. Findings should therefore be interpreted as reflecting a range of experiences within broad language groups rather than representing specific cultural or national populations.

While this study did not systematically examine differences based on age or parity, we observed that younger participants were more likely to have higher levels of formal education, as well as stronger English language and digital skills, which appeared to facilitate navigation of services. However, these patterns were not formally analysed. As such, the perspectives of women experiencing greater marginalisation – including those with lower health literacy, limited formal education, or more acute social disadvantage – may be underrepresented.

A further limitation concerns the multilingual data collection and transcription process. Non-English sessions were conducted with real-time interpreter support, and transcripts were produced from the interpreter’s English translation rather than from original-language audio recordings. While literal translation was prioritised and culturally significant terms were reviewed with the Advisory Group, this process means that some nuance, culturally specific meaning, or colloquial expression may have been lost prior to analysis.

Our findings relating to digital resources should also be interpreted within the context of the interview design. As part of the interview guide, participants were shown and asked to comment on several pre-selected online perinatal mental health resources, which likely foregrounded discussion of digital platforms and contributed to the prominence of themes related to trust, navigation, accessibility, and perceived usefulness. While this approach enabled exploration of participants’ reflections on existing resources, it may also have shaped the emphasis placed on digital health within the findings. Nonetheless, participants contextualised these discussions within broader perinatal mental health care pathways, highlighting strategies, barriers, and supports across digital, community, and healthcare settings.

Furthermore, while this study explored women’s emotional wellbeing and sources of stress, it did not directly examine experiences of domestic and family violence. The limited reporting of such experiences in our data may reflect the sensitivity of the topic, the group-based data collection format, and the absence of direct questioning. Given the well-established links between domestic and family violence and perinatal mental health [76, 77], future research would benefit from partnerships with specialist services to more safely explore these intersections.

## Conclusion

This study explored how women from Chinese-, Arabic-, and Indian-language speaking backgrounds in Victoria, Australia, navigated perinatal mental health care across clinical, community, and digital settings. Within this qualitative sample — largely tertiary-educated, predominantly metropolitan-based, and recruited through community networks — participants described a complex interplay of cultural and social norms, stigma, systemic healthcare barriers, and migration-related disruptions that appeared to influence their perinatal mental health experiences and care navigation. While family and peer networks were frequently described as important sources of support, many participants recounted navigating fragmented services and conflicting cultural expectations without clear guidance or safe spaces for disclosure. Participants also identified social supports through family, peer connections, professionally facilitated settings, and digital platforms, as contributing to their sense of wellbeing by providing reassurance, opportunities for knowledge exchange, and social connection.

Digital platforms were described by some participants as accessible and immediate sources of support, particularly among women encountering barriers to formal services. However, participants were shown five pre-selected digital resources prior to interview, which likely shaped the prominence of discussion relating to digital platforms; findings regarding digital tool use should therefore be interpreted within this methodological context. Engagement with digital resources also appeared to vary according to trust, cultural relevance, language accessibility, and levels of digital literacy, which influenced their perceived usefulness for some participants. Within the limits of this qualitative study, findings suggest that digital tools may represent one component of broader perinatal mental health support pathways for some migrant women, although their perceived value appeared to depend on cultural responsiveness, language accessibility, and meaningful integration with formal care pathways. Overall, these findings highlight the potential value of equity-focused and co-designed approaches developed in partnership with women from migrant communities to support culturally relevant and accessible perinatal mental health care across clinical, community, and digital settings.

## Supplementary Information

Supplementary Material 1.

## Data Availability

The data used and/or analysed during the current study are available upon reasonable request. Access to the data may be granted to those who provide a justified request for research purposes. To request access, please contact areni.altun@monash.edu.

## Abbreviations

CCI: Consumer and Community Involvement
COREQ: Consolidated Criteria for Reporting Qualitative Research
GP: General Practitioner
MCH: Maternal Child Health
NAATI: National Accreditation Authority for Translators and Interpreters
WHRTN: National Women’s Health Research, Translation and Impact Network
