# Supplementary material for: Navigating perinatal mental health care: migrant women’s experiences of support pathways and the role of digital resources
Source: Arch Public Health. 2026 Jun 11;84:175. doi: 10.1186/s13690-026-01989-x (PMC13439841; doi:10.1186/s13690-026-01989-x)
Supplement: Supplementary file 1 [file 13690_2026_1989_MOESM1_ESM.docx]

# Interview Guide

*“Thank you for agreeing to take part in our study. We are interested in better understanding the experiences of migrant and refugee women across Australia and how they navigate online resources for their mental health needs before, during and after pregnancy. An example of what we mean by online resources is websites, online chat groups or apps on your mobile phone. I understand that talking about mental health is not common in some cultures but the more open you are about these topics, the more it will help us understand what support can be offered to migrant and refugee women. Before we begin I would like to say that pregnancy, parenting and having a baby is a significant life event and it is common that this can feel stressful and overwhelming. But there are resources available and this is how we want to help. Signs and symptoms women may experience during or after pregnancy might include tiredness, sleep problems, anger, difficulty eating, less enjoyment, guilt, sadness and worry and so on - but this may differ from person to person and early stages are often not connected to mental health issues. If you would prefer to skip any question, please let me know. The interview will take approximately 60-90 minutes and we can pause at any time if you need to. The interview will be recorded, is this OK? Do you have any questions about the interview or the consent form before we start?"*

1. Prior to coming to this interview, I had sent through a range of websites (COPE, PANDA, Raisingchildren, RAMA, MCWH). Can you tell me what you thought of these websites?
   a. What has been done well?
   b. What do you think could be improved?

***Prompts:*** *Navigability, content, language, images, health literacy etc.*

c. Which one do you feel was most helpful and why?

***Prompts:*** *Images, written information, colours etc.*

2. Do you think there are any cultural beliefs that influence how women access online information or services? Any barriers/any cultural factors that help?
3. If you were a woman arriving to Australia from overseas, with only limited English language, and you were struggling after giving birth, would you look for an online resource to support you?
a. Why/why not?
b. How do you think women might find an online resource?

***Prompts:*** *Health professionals/friends/family/social media etc.*

4. If a woman you knew had just given birth and was going through a difficult time, what do you think could support her?
   a. Do you think any of the online resources that were shared with you might help her during this time? Which ones? Why or why not?
5. What do you think can best help migrant women with their mental wellbeing before, during and after pregnancy?
   a. Do you think online resources would work? Why or why not?

***Prompts:*** *is privacy a concern? Do you feel safe? Do women have their own device? Is digital literacy an issue?*

6. What gaps, if any, do you feel there are in the mental health support given to women before, during and after pregnancy?
7. Lastly, is there anything you would like to tell me or share, that I have not asked?
